# Radio-frequency ablation vs. open surgery in the treatment of varicose veins - a comparative study

**DOI:** 10.1186/1753-6561-9-S1-A8

**Published:** 2015-01-14

**Authors:** W Tashkandi, T Aherne, J Byrne, D Monoley

**Affiliations:** 1 Royal College of Surgeons in Ireland, 123 St. Stephen’s Green, Dublin 2, Ireland; 2 Department of Vascular Surgery, Beaumont Hospital, Dublin 9. Ireland

## Background

Varicose veins are common and often debilitating. Radio-frequency ablation (RFA) has emerged as a minimally invasive alternative to traditional open venous ligation surgery. It has been shown to reduce peri-operative morbidity and improve quality of life scores. Aim: The aim of this study was to directly compare RFA and open sapheno-femoral ligation.

## Methods

This was a single centre retrospective cohort study. All patients with confirmed sapheno-femoral junctional incompetence who underwent surgical management treatment between January 2011 and December 2012 were included. Medical charts and computer records were reviewed. Radiological success, choice of anaesthesia and hospital length of stay was documented. Procedural cost was also calculated. A focused cohort analysis was undertaken to compare the initial 50 RFA procedures performed with the last 50. This allowed for departmental learning curve assessment over a 13-month period.

## Results

During the study period 298 patients underwent surgical intervention. A total of 204 patients underwent RFA. Sixty-six percent of all patients were female. RFA was associated with a reduction in the requirement for general anesthesia (41% v 100%, P=0.000), overnight hospital stay (22% v 82%, P=0.000) and pre-operative blood tests (5% v 38%, P=0.000) when compared with open ligation. The overall success rate for RFA was 98%. No significant inter-group difference was noted for 30-day readmission (p=0.203). The cost of open surgery was significantly less than RFA (€996 v. €734, P=0.000). Subgroup analysis with regard RFA identified a reduction in cost (€1024 v. €971, P=0.003) as well as hospital overnight stays (10% v 36%) with an increase in the use of intravenous sedation as opposed to general anaesthetic (18% v 60%) over a 13-month period.

## Conclusion

RFA is a viable alternative to open repair, requiring less invasive anaesthesia, fewer laboratory tests and reducing hospital length of stay. However, it is associated with a higher financial cost.

